# Supplementary material for: Fragmentation by major dams and implications for the future viability of platypus populations
Source: Commun Biol. 2022 Nov 3;5:1127. doi: 10.1038/s42003-022-04038-9 (PMC9633709; doi:10.1038/s42003-022-04038-9)
Supplement: Supplementary file 2 — Supplementary Information [file 42003_2022_4038_MOESM2_ESM.pdf]

Fragmentation by major dams and implications for the future viability of platypus populations

Jose L. Mijangos[1,2], Gilad Bino[3], Tahneal Hawke[3], Stephen H. Kolomyjec[4], Richard T. Kingsford[3], Harvinder Sidhu[1], Tom Grant[3], Jenna Day[5], Kimberly N. Dias[5], Jaime Gongora[5] and William B. Sherwin[6].

[1] School of Science, UNSW, Canberra, Australia.

[2] Centre for Conservation Ecology and Genomics, Institute for Applied Ecology, University of Canberra, Canberra, Australia.

[3] Centre for Ecosystem Science, School of Biological, Earth and Environmental Sciences, UNSW, Sydney, Australia.

[4] College of Science and the Environment, Lake Superior State University, Sault Sainte Marie, USA.

[5] Sydney School of Veterinary Science, Faculty of Science, The University of Sydney, Sydney, Australia.

[6] Evolution & Ecology Research Centre, UNSW, Sydney, Australia.

Corresponding author: Jose L. Mijangos. E-mail: luis.mijangos@gmail.com

SUPPLEMENTARY INFORMATION

Filtering process of molecular markers

We first describe the filters applied to the entire SNP dataset, then those applied for analyses of variation between groups, and finally the filters applied to analyses of variation within groups. The filtering was performed using the R[1] package *dartR*[2].

Filters applied to the entire SNP dataset. Because filtering for Hardy-Weinberg proportions requires the delimitation of populations or groups, we assigned individuals *a priori* into groups, based on the river that individuals were sampled and whether they were sampled below or above major dams. To reduce genotyping errors that might have arisen during library preparation and SNP calling[3], we discarded sites that had a reproducibility of less than 100% (RepAvg). We also discarded sites if the read had more than one SNP; or showed a

significant departure from Hardy-Weinberg proportions within any one group after Bonferroni correction with a p-value of less than 0.05; or was not mapped to a chromosome and had a BLAST alignment E-value of more than 1e-20 .

Filters applied before analyses of genetic variation between groups in the SNP dataset. We discarded sites with more than 10% of missing data. By discarding sites based on minor allele frequency (*i.e.*, proportion, MAF), there is the potential to alter subsequent analyses[4]. Therefore, we discarded sites with a minor allele count (MAC) of less than three. Because physical linkage between sites can affect analyses of genetic structure[5], we removed one of two sites if they were in linkage disequilibrium (LD). For this, we used a threshold of the LD statistic r^2 [6] of > 0.2. Then the SNP with lower polymorphic information content (AvgPIC) was discarded. Selectively neutral sites are better suited to infer population dynamics, such as dispersal, than sites under selection because they allow separation from potential confounding factors arising from natural selection[7]. Consequently, we removed sites located within coding regions using the GFF (General Feature Format; GCF_004115215.1_mOrnAna1.p.v1_genomic.gff.gz) file retrieved from NCBI[8]. Despite 3rd position SNPs usually being silent (*i.e.*, synonymous mutations), we also filtered out these SNPs for two reasons. Firstly, recent research suggests that synonymous mutations also affect fitness[9]. Secondly, natural selection can affect not only genetic variation with direct consequences on fitness but can also affect adjacent neutral genetic variation due to genetic linkage[10].

Filters applied before analyses of genetic variation within groups in the SNP dataset. When sites with missing data are not removed, observed (*Ho*) and expected heterozygosity (*He*) estimates diverge[11]; accordingly, we removed sites with missing data. *He* is generally lower for SNPs with rare alleles than for SNPs with common alleles[11]. Therefore we did not filter out sites using minor allele count (MAC). Because filtering out sites based on linkage disequilibrium results in a decrease of rare and monomorphic alleles and excess of the common alleles and therefore biases estimates of *He*[12], we did not remove sites that were in linkage disequilibrium. Estimates of *He* should reflect as much as possible genome-wide patterns[13]. Therefore we kept sites located outside and inside coding regions. We removed sites in sex chromosomes for two reasons. Firstly, *He* in sex chromosomes differs from *He* in

autosomes because sex chromosomes occur in different proportions in males and females[14]. Secondly, the platypus has five pairs of sex chromosomes[15], which will bias the overall estimation of *He*. Finally, relatedness analyses using the R package *related*[16] were performed to identify any recaptures not identified or mislabelling in the field or the laboratory.

Investigation of contrasting genetic patterns relative to samples collected in the same river

Two samples, each collected in a different river (V30 in Ovens and V32 in Mitta Mitta), showed contrasting genetic patterns relative to samples collected in the same river (Supplementary Figure 1). Relatedness analyses performed in the R package *related*[16] revealed these two samples had closer relatives in the opposite river (Supplementary Table 1). Additionally, the locations of these two samples were separated by 46 Km, steep mountainous terrain, and a river system. Under these conditions, we considered that dispersal events were unlikely and concluded that samples were mislabelled and therefore assigned them to the presumed correct river and site.

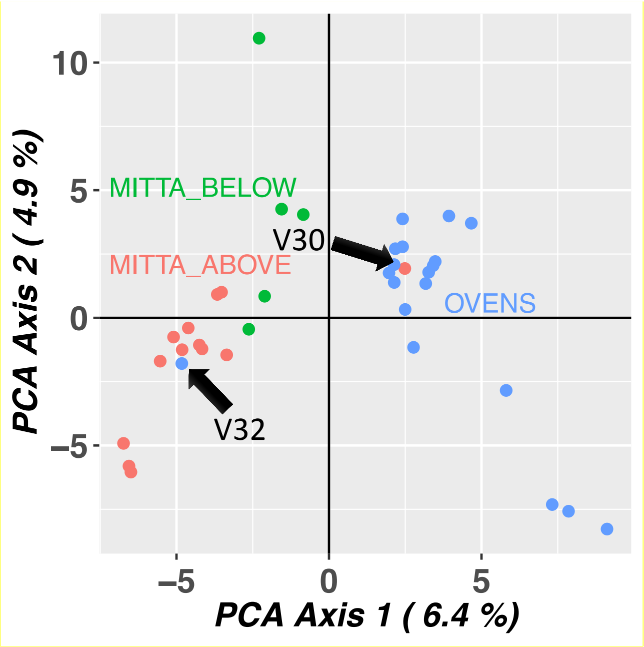

Supplementary Figure 1 | Preliminary analysis to check data integrity: Principal component analysis (PCA) based on SNP data. Figure shows that the individuals V30 and V32 (indicated by arrows), each collected in a different river, showed contrasting genetic patterns relative to individuals collected in the same river.

An alternative explanation of these contrasting patterns would be two dispersing juvenile platypuses swimming upstream until they reach the upper headwaters of their home catchments, then move across land/uphill until they reach the catchment boundaries, then travel downhill until they reach a gully leading to the headwaters of adjoining catchments, then re-enter the water and swim downstream until they find suitable vacant habitat to occupy.

Supplementary Table 1 | Preliminary analysis to check data integrity. Results of relatedness analyses, based on SNP data, performed in the R package *related*[16] to identify any recapture not identified or mislabelling either in the field or the laboratory. Individual codes are as in Supplementary Table 2.

Ind 1	Population	Ind 2	Population	Coefficient of relatedness
V30	OVENS	V1	MITTA_ABOVE	0.2431
V30	OVENS	V5	MITTA_ABOVE	0.2289
V30	OVENS	V33	MITTA_ABOVE	0.1778
V30	OVENS	V25	MITTA_ABOVE	0.1566
V30	OVENS	V2	MITTA_ABOVE	0.1423
V32	MITTA_ABOVE	V10	OVENS	0.1695
V32	MITTA_ABOVE	V15_2	OVENS	0.1612
V32	MITTA_ABOVE	V14	OVENS	0.1381
V32	MITTA_ABOVE	V19	OVENS	0.1324
V32	MITTA_ABOVE	V9	OVENS	0.1215
T3	TENTERFIELD	T5	TENTERFIELD	0.9902
T28	TENTERFIELD	T42	TENTERFIELD	0.9799

Supplementary Table 2 | General information in the SNP dataset per individual.

ID	Group	Sex	Age class	Weight	Latitude	Longitude
E65	Eucumbene above	Female	Juvenile	0.58	-35.918995	148.540494
E68	Eucumbene above	Female	Adult	0.93	-35.887646	148.51589
E69	Eucumbene above	Female	Juvenile	0.56	-35.887646	148.51589
E70	Eucumbene above	Male	Adult	1.36	-35.887646	148.51589
E1	Eucumbene below	Male	Sub-adult	1.12	-36.164286	148.622989
E10	Eucumbene below	Female	Adult	0.94	-36.222028	148.632604
E11	Eucumbene below	Female	Adult	0.84	-36.253799	148.620099
E12	Eucumbene below	Female	Adult	0.82	-36.253799	148.620099
E18	Eucumbene below	Male	Adult	1.27	-36.181475	148.633916
E19	Eucumbene below	Male	Adult	1.30	-36.183226	148.634856
E2	Eucumbene below	Female	Adult	NA	-36.164286	148.622989
E20	Eucumbene below	Male	Sub-adult	1.25	-36.183226	148.634856
E28	Eucumbene below	Female	Juvenile	0.75	-36.180689	148.634867
E29	Eucumbene below	Female	Adult	0.91	-36.180689	148.634867
E3	Eucumbene below	Female	Adult	0.87	-36.164286	148.622989
E4	Eucumbene below	Female	Adult	0.83	-36.181475	148.633916
E5	Eucumbene below	Male	Adult	1.58	-36.181475	148.633916
E6	Eucumbene below	Male	Adult	1.48	-36.180689	148.634867
E63	Eucumbene below	Female	Adult	0.78	-36.180689	148.634867
E64	Eucumbene below	Female	Juvenile	0.74	-36.181475	148.633916
E7	Eucumbene below	Male	Adult	1.56	-36.181475	148.633916
E74	Eucumbene below	Male	Adult	1.47	-36.180689	148.634867
E8	Eucumbene below	Female	Adult	0.77	-36.181475	148.633916
E9	Eucumbene below	Male	Adult	1.09	-36.222028	148.632604
V1	Mitta above	Female	Juvenile	NA	-36.908784	147.622772
V2	Mitta above	Female	Juvenile	0.59	-36.908784	147.622772
V21	Mitta above	Male	Adult	0.93	-36.946087	147.607521
V22	Mitta above	Male	Adult	1.14	-36.946087	147.607521
V23	Mitta above	Male	Adult	1.18	-36.891481	147.631409
V24	Mitta above	Male	Adult	1.28	-36.891481	147.631409
V25	Mitta above	Female	Juvenile	0.51	-36.891481	147.631409

V3	Mitta above	Male	Juvenile	NA	-36.806872	147.66193
V30	Mitta above	Male	Adult	1.68	-36.699684	146.9096
V31	Mitta above	Female	Juvenile	0.46	-36.891481	147.631409
V33	Mitta above	Female	Adult	0.76	-36.891481	147.631409
V4	Mitta above	Male	Juvenile	NA	-36.851574	147.637379
V5	Mitta above	Male	Juvenile	NA	-36.891481	147.631409
V20	Mitta below	Male	Juvenile	0.82	-36.508674	147.411724
V35	Mitta below	Male	Adult	1.58	-36.508228	147.412276
V36	Mitta below	Male	Adult	1.64	-36.514876	147.374988
V37	Mitta below	Male	Adult	1.36	-36.51879	147.377085
V10	Ovens	Female	Adult	0.93	-36.725589	146.963083
V11	Ovens	Female	Adult	0.88	-36.725589	146.963083
V12	Ovens	Female	Adult	0.99	-36.725589	146.963083
V13	Ovens	Male	Juvenile	0.90	-36.820253	147.072079
V14	Ovens	Female	Adult	1.02	-36.717882	146.945064
V15_1	Ovens	Female	Adult	1.27	-36.717882	146.945064
V15_2	Ovens	Male	Adult	1.46	-36.803133	147.057653
V17	Ovens	Female	Juvenile	0.78	-36.629973	146.821417
V18	Ovens	Male	Juvenile	0.76	-36.699684	146.9096
V19	Ovens	Female	Adult	0.87	-36.699684	146.9096
V26	Ovens	Male	Juvenile	1.02	-36.638183	146.851507
V27	Ovens	Female	Juvenile	0.82	-36.638183	146.851507
V28	Ovens	Female	Adult	1.03	-36.656708	146.856924
V29	Ovens	Male	Adult	1.34	-36.738527	146.975783
V32	Ovens	Male	Adult	1.36	-36.891481	147.631409
V6	Ovens	Female	Adult	NA	-36.699684	146.9096
V7	Ovens	Female	Adult	NA	-36.699684	146.9096
V8	Ovens	Female	Adult	NA	-36.699684	146.9096
V9	Ovens	Male	Adult	1.56	-36.745153	147.016953
SUS19	Severn above	Male	Adult	0.93	-29.474119	151.485041
SUS20	Severn above	Female	Adult	1.34	-29.474119	151.485041
SUS21	Severn above	Female	Adult	1.13	-29.474119	151.485041
SUS22	Severn above	Male	Adult	1.84	-29.49832	151.562924

SUS23	Severn above	Male	Adult	1.80	-29.493532	151.543319
SUS24	Severn above	Male	Juvenile	1.15	-29.493532	151.543319
SUS25	Severn above	Female	Adult	1.22	-29.506811	151.589001
SUS26	Severn above	Male	Adult	1.86	-29.506811	151.589001
SUS27	Severn above	Female	Adult	1.16	-29.506811	151.589001
SUS28	Severn above	Female	Adult	1.23	-29.506811	151.589001
SUS29	Severn above	Male	Adult	1.47	-29.506811	151.589001
SUS30	Severn above	Male	Adult	1.74	-29.506811	151.589001
SUS31	Severn above	Male	Adult	2.01	-29.502849	151.611012
SUS32	Severn above	Male	Adult	1.73	-29.463007	151.478227
SUS33	Severn above	Female	Adult	1.21	-29.463007	151.478227
SUS34	Severn above	Male	Adult	1.84	-29.463007	151.478227
SUS35	Severn above	Female	Adult	1.22	-29.463007	151.478227
SUS36	Severn above	Female	Adult	1.28	-29.456671	151.464321
SUS37	Severn above	Male	Adult	1.64	-29.456671	151.464321
SUS38	Severn above	Male	Adult	1.45	-29.456671	151.464321
SUS39	Severn above	Female	Juvenile	0.96	-29.456671	151.464321
SUS40	Severn above	Male	Adult	1.84	-29.456671	151.464321
SUS41	Severn above	Male	Adult	2.08	-29.456671	151.464321
SDS10	Severn below	Male	Adult	1.64	-29.179456	151.111784
SDS11	Severn below	Female	Adult	1.27	-29.179456	151.111784
SDS12	Severn below	Male	Adult	1.68	-29.179456	151.111784
SDS13	Severn below	Male	Adult	1.43	-29.303547	151.138189
SDS14	Severn below	Male	Adult	1.83	-29.302878	151.124002
SDS15	Severn below	Male	Juvenile	1.05	-29.302878	151.124002
SDS16	Severn below	Male	Adult	2.11	-29.249669	151.133357
SDS17	Severn below	Female	Adult	1.24	-29.249669	151.133357
SDS18	Severn below	Female	Adult	1.22	-29.249669	151.133357
SDS2	Severn below	Male	Adult	1.66	-29.296528	151.144227
SDS3	Severn below	Female	Adult	1.07	-29.296528	151.144227
SDS4	Severn below	Female	Adult	1.24	-29.296528	151.144227
SDS5	Severn below	Female	Adult	1.39	-29.189063	151.132304
SDS6	Severn below	Male	Adult	1.80	-29.189063	151.132304

SDS7	Severn below	Male	Adult	1.77	-29.232346	151.114818
SDS8	Severn below	Male	Adult	0.84	-29.232346	151.114818
SDS9	Severn below	Female	Adult	1.34	-29.27808	151.117899
E100	Snowy	Male	Adult	1.03	-36.501988	148.831949
E13	Snowy	Male	Adult	1.11	-36.467166	148.693223
E14	Snowy	Male	Adult	1.53	-36.467166	148.693223
E21	Snowy	Female	Adult	0.78	-36.447367	148.648261
E22	Snowy	Female	Adult	0.78	-36.447367	148.648261
E23	Snowy	Female	Adult	0.86	-36.480859	148.774219
E24	Snowy	Female	Adult	0.85	-36.480859	148.774219
E25	Snowy	Male	Adult	1.55	-36.480859	148.774219
E26	Snowy	Female	Adult	0.93	-36.447367	148.648261
E27	Snowy	Female	Adult	0.80	-36.447367	148.648261
E33	Snowy	Female	Adult	1.00	-36.480859	148.774219
E34	Snowy	Male	Adult	1.45	-36.480859	148.774219
E36	Snowy	Female	Juvenile	0.45	-36.447412	148.643942
E37	Snowy	Male	Adult	1.29	-36.447412	148.643942
E38	Snowy	Male	Adult	1.52	-36.447412	148.643942
E39	Snowy	Female	Adult	0.93	-36.447412	148.643942
E40	Snowy	Male	Adult	1.21	-36.439499	148.63292
E41	Snowy	Male	Juvenile	0.52	-36.439499	148.63292
E42	Snowy	Male	Juvenile	0.47	-36.443192	148.636579
E43	Snowy	Female	Adult	0.81	-36.443192	148.636579
E44	Snowy	Female	Juvenile	0.46	-36.443192	148.636579
E45	Snowy	Male	Adult	1.42	-36.443192	148.636579
E46	Snowy	Female	Adult	NA	-36.443192	148.636579
E47	Snowy	Female	Adult	0.85	-36.446414	148.652903
E48	Snowy	Female	Adult	0.89	-36.446267	148.661103
E49	Snowy	Male	Adult	1.40	-36.446267	148.661103
E50	Snowy	Male	Adult	1.33	-36.446267	148.661103
E51	Snowy	Female	Adult	0.89	-36.446267	148.661103
E52	Snowy	Female	Adult	0.72	-36.446414	148.652903
E53	Snowy	Female	Adult	0.89	-36.447367	148.648261

E54	Snowy	Male	Adult	1.48	-36.447367	148.648261
E57	Snowy	Female	Adult	0.71	-36.480859	148.774219
E58	Snowy	Male	Adult	1.76	-36.480859	148.774219
E59	Snowy	Female	Juvenile	0.48	-36.480859	148.774219
E67	Snowy	Male	Adult	1.47	-36.452387	148.677242
E71	Snowy	Male	Adult	1.59	-36.480859	148.774219
E72	Snowy	Female	Adult	0.96	-36.480859	148.774219
E73	Snowy	Female	Adult	0.94	-36.480859	148.774219
E79	Snowy	Male	Adult	NA	-36.501988	148.831949
E80	Snowy	Male	Adult	1.23	-36.501988	148.831949
E81	Snowy	Female	Adult	NA	-36.501988	148.831949
E82	Snowy	Male	Adult	1.37	-36.501988	148.831949
E83	Snowy	Female	Adult	1.03	-36.501988	148.831949
E84	Snowy	Male	Adult	1.53	-36.501988	148.831949
E85	Snowy	Male	Adult	1.67	-36.46894	148.722478
E86	Snowy	Female	Adult	0.72	-36.46894	148.722478
E87	Snowy	Female	Adult	0.81	-36.46894	148.722478
E88	Snowy	Male	Adult	NA	-36.46894	148.722478
E89	Snowy	Male	Adult	1.18	-36.46894	148.722478
E93	Snowy	Male	Sub-adult	0.64	-36.439499	148.63292
E94	Snowy	Male	Adult	NA	-36.480859	148.774219
E95	Snowy	Female	Adult	0.70	-36.501988	148.831949
E96	Snowy	Male	Sub-adult	1.10	-36.501988	148.831949
E97	Snowy	Male	Adult	NA	-36.447367	148.648261
E98	Snowy	Male	Adult	1.15	-36.447367	148.648261
E99	Snowy	Female	Adult	0.68	-36.480859	148.774219
T1	Tenterfield	Female	Juvenile	0.90	-29.036629	151.820534
T10	Tenterfield	Female	Juvenile	0.57	-29.035987	151.759721
T11	Tenterfield	Female	Juvenile	0.82	-29.035987	151.759721
T12	Tenterfield	Female	Adult	1.05	-29.035987	151.759721
T13	Tenterfield	Female	Adult	0.98	-28.984905	151.951568
T14	Tenterfield	Male	Adult	1.60	-28.984905	151.951568
T15	Tenterfield	Female	Juvenile	0.82	-28.984905	151.951568

T16	Tenterfield	Female	Adult	0.91	-28.984905	151.951568
T17	Tenterfield	Male	Juvenile	0.85	-28.984905	151.951568
T18	Tenterfield	Female	Juvenile	0.67	-29.022531	151.86925
T19	Tenterfield	Male	Adult	1.79	-29.022531	151.86925
T2	Tenterfield	Male	Adult	1.79	-29.030611	151.851523
T20	Tenterfield	Male	Adult	1.69	-29.002064	151.995101
T21	Tenterfield	Female	Adult	1.02	-29.002064	151.995101
T22	Tenterfield	Male	Juvenile	1.07	-29.002064	151.995101
T23	Tenterfield	Male	Adult	1.58	-29.030611	151.851523
T24	Tenterfield	Male	Adult	1.55	-29.014065	151.864932
T25	Tenterfield	Male	Adult	1.51	-29.014065	151.864932
T26	Tenterfield	Male	Adult	1.50	-28.989203	151.701152
T27	Tenterfield	Female	Adult	0.91	-28.989203	151.701152
T28	Tenterfield	Female	Juvenile	0.79	-29.026713	151.746975
T29	Tenterfield	Female	Adult	0.98	-29.026713	151.742656
T30	Tenterfield	Female	Adult	0.94	-29.031212	151.742656
T31	Tenterfield	Female	Adult	0.79	-29.031212	151.742656
T32	Tenterfield	Female	Adult	1.56	-29.031212	151.742656
T33	Tenterfield	Male	Adult	1.39	-29.027057	151.724683
T34	Tenterfield	Female	Adult	0.83	-29.027057	151.724683
T35	Tenterfield	Male	Adult	1.62	-29.027057	151.724683
T36	Tenterfield	Female	Adult	1.00	-29.027057	151.724683
T38	Tenterfield	Female	Adult	1.01	-29.014825	151.722949
T39	Tenterfield	Female	Adult	0.98	-29.014825	151.722949
T4	Tenterfield	Male	Juvenile	0.88	-29.011746	151.86722
T40	Tenterfield	Male	Juvenile	0.82	-28.959487	151.544385
T41	Tenterfield	Female	Adult	1.08	-28.959487	151.544385
T5	Tenterfield	Female	Juvenile	0.62	-29.011746	151.86722
T6	Tenterfield	Female	Juvenile	0.93	-29.011746	151.86722
T7	Tenterfield	Female	Juvenile	0.91	-29.035987	151.759721
T8	Tenterfield	Female	Adult	1.23	-29.035987	151.759721
T9	Tenterfield	Male	Juvenile	0.11	-29.035987	151.759721
E15	Thredbo	Female	Adult	0.61	-36.44681	148.424506

E16	Thredbo	Male	Adult	1.10	-36.44681	148.424506
E17	Thredbo	Female	Adult	0.70	-36.44681	148.424506
E30	Thredbo	Male	Adult	1.20	-36.410407	148.495869
E31	Thredbo	Female	Adult	0.91	-36.410407	148.495869
E35	Thredbo	Male	Adult	1.33	-36.44681	148.424506
E55	Thredbo	Female	Adult	0.63	-36.410407	148.495869
E56	Thredbo	Female	Adult	0.92	-36.410407	148.495869
E60	Thredbo	Female	Juvenile	0.50	-36.44681	148.424506
E61	Thredbo	Male	Adult	0.90	-36.44681	148.424506
E62	Thredbo	Male	Adult	1.60	-36.44681	148.424506
E66	Thredbo	Female	Juvenile	0.40	-36.467726	148.373502
E75	Thredbo	Male	Juvenile	0.66	-36.369731	148.589586
E76	Thredbo	Female	Adult	0.95	-36.369731	148.589586
E77	Thredbo	Female	Juvenile	0.44	-36.376912	148.583204
E78	Thredbo	Male	Juvenile	0.76	-36.376912	148.583204
E90	Thredbo	Female	Juvenile	NA	-36.438257	148.443454
E91	Thredbo	Male	Adult	1.50	-36.497628	148.316844
E92	Thredbo	Male	Adult	0.91	-36.497628	148.316844

Supplementary Table 3 | Genetic differentiation using Mutual information (MI[17]) and Jost's D[18] between rivers in different connectivity scenarios.

Region	River 1	River 2	*MI*	SE	*Jost's D*	SE	Connectivity scenario
Border Rivers	Tenterfield	Severn above dam	0.011	0.000	0.009	0.001	Separated by a river system
	Tenterfield	Severn below dam	0.012	0.000	0.011	0.001	Separated by a river system
	Severn below dam	Severn above dam	0.011	0.000	0.008	0.001	Separated by dam for 47 years (*Circa* 1969)*
	Tenterfield above	Tenterfield below	0.006	0.000	0.001	0.000	No dam
Upper Murray Rivers	Ovens	Mitta-Mitta above dam	0.013	0.000	0.008	0.001	Contiguous river systems
	Ovens	Mitta-Mitta below dam	0.011	0.000	0.006	0.001	Contiguous river systems
	Mitta-Mitta above dam	Mitta-Mitta below dam	0.013	0.000	0.004	0.001	Separated by dam for 39 years (*Circa* 1979)
	Ovens above	Ovens below	0.010	0.000	0.000	0.001	No dam
Snowy Rivers	Snowy	Thredbo	0.005	0.000	0.003	0.000	Separated by dam for 50 years (*Circa* 1967)
	Snowy	Eucumbene above dam	0.005	0.000	0.006	0.001	Separated by dam for 59 years (*Circa* 1958)
	Snowy	Eucumbene below dam	0.007	0.000	0.006	0.001	Separated by dam for 50 years (*Circa* 1967)
	Thredbo	Eucumbene above dam	0.011	0.000	0.006	0.001	Separated by dam for 59 years (*Circa* 1958)
	Thredbo	Eucumbene below dam	0.009	0.000	0.004	0.001	Separated by lake for 50 years (*Circa* 1967)
	Eucumbene above dam	Eucumbene below dam	0.011	0.000	0.008	0.001	Separated by dam for 59 years (*Circa* 1958)
Central NSW Rivers	Wingecarribee**	Nepean above dam	0.084	0.012	0.142	0.048	Contiguous river systems
	Wingecarribee**	Nepean below dam	0.066	0.010	0.135	0.043	Contiguous river systems
	Nepean above dam**	Nepean below dam	0.187	0.029	0.133	0.053	Separated by dam for 74 years (*Circa* 1935)
	Wingecarribee above**	Wingecarribee below	0.082	0.015	0.046	0.023	No dam

SE - standard error.
***** Pindari Dam. The height of the dam wall was doubled from 45m to 85m in 1995.
****** Microsatellite data

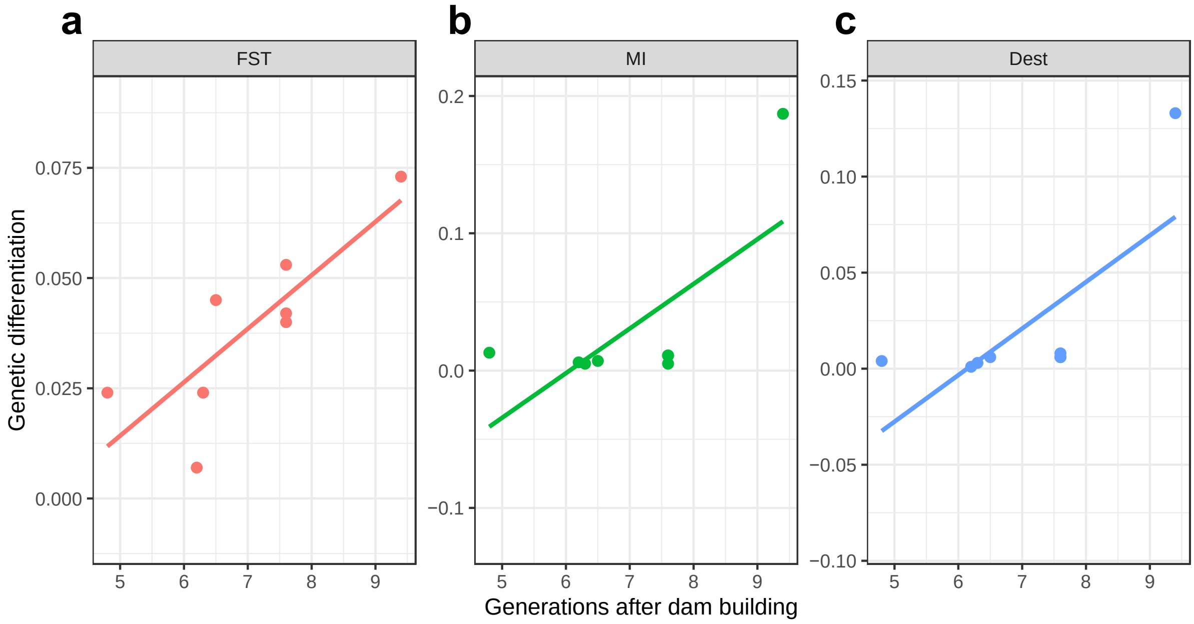

Supplementary Figure 2 | Genetic differentiation against dam age. Relationship between genetic differentiation using **a** F_{ST}, **b** Mutual information (MI[17]) and **c** Jost's D[18] (Dest) between platypus groups separated by major dams (n = 8 major dams) and the number of platypus generations (7.9 years; Pacifici et al., 2013) since the building of the dam.

Supplementary Table 4 | Summary statistics of linear regression between genetic differentiation measures and dam age.

	F_{ST}	Mutual Information	Jost's D
**r.squared**	0.670	0.493	0.533
**adj.r.squared**	0.615	0.408	0.455
**sigma**	0.847	1.051	1.009
**statistic**	12.195	5.830	6.837
**p.value**	0.013	0.052	0.040
**df**	1	1	1
**logLik**	-8.874	-10.597	-10.270
**AIC**	23.749	27.193	26.539
**BIC**	23.987	27.431	26.777
**deviance**	4.307	6.624	6.104
**df.residual**	6	6	6
**nobs**	8	8	8

References

1. *R Core Team. R: A language and environment for statistical computing. R Foundation for Statistical Computing, Vienna, Austria* **URL** https://www.R-project.org/*, (2021).*

2. *Mijangos JL, Gruber B, Berry O, Pacioni C, Georges A. dartR v2: an accessible genetic analysis platform for conservation, ecology, and agriculture. Methods in Ecology and Evolution, (2022).*

3. *O'Leary SJ, Puritz JB, Willis SC, Hollenbeck CM, Portnoy DS. These aren't the loci you'e looking for: Principles of effective SNP filtering for molecular ecologists. Mol Ecol* ***27****, 3193-3206 (2018).*

4. *Linck E, Battey CJ. Minor allele frequency thresholds strongly affect population structure inference with genomic data sets. Mol Ecol Resour* ***19****, 639-647 (2019).*

5. *Abdellaoui A, et al. Population structure, migration, and diversifying selection in the Netherlands. European journal of human genetics* ***21****, 1277-1285 (2013).*

6. *Hill WG, Robertson A. Linkage disequilibrium in finite populations. Theoretical and applied genetics* ***38****, 226-231 (1968).*

7. *Holderegger R, Wagner HH. Landscape genetics. Bioscience* ***58****, 199-207 (2008).*

8. *Database resources of the National Center for Biotechnology Information. Nucleic Acids Res* ***44****, D7-19 (2016).*

9. *Lebeuf-Taylor E, McCloskey N, Bailey SF, Hinz A, Kassen R. The distribution of fitness effects among synonymous mutations in a gene under directional selection. Elife **8**, e45952 (2019).*

10. *Smith JM, Haigh J. The hitch-hiking effect of a favourable gene. Genetical research **23**, 23-35 (1974).*

11. *Schmidt TL, Jasper M, Weeks AR, Hoffmann AA. Unbiased population heterozygosity estimates from genome-wide sequence data. Methods in Ecology and Evolution, (2020).*

12. *Dementieva N, et al. Assessing the effects of rare alleles and linkage disequilibrium on estimates of genetic diversity in the chicken populations. Animal **15**, 100171 (2021).*

13. *Miller J, et al. Estimating genome-wide heterozygosity: effects of demographic history and marker type. Heredity **112**, 240-247 (2014).*

14. *Schaffner SF. The X chromosome in population genetics. Nat Rev Genet **5**, 43-51 (2004).*

15. *Veyrunes F, et al. Bird-like sex chromosomes of platypus imply recent origin of mammal sex chromosomes. Genome research **18**, 965-973 (2008).*

16. *Pew J, Muir PH, Wang J, Frasier TR. related: an R package for analysing pairwise relatedness from codominant molecular markers. Mol Ecol Resour **15**, 557-561 (2015).*

17. *Sherwin WB, Chao A, Jost L, Smouse PE. Information theory broadens the spectrum of molecular ecology and evolution. Trends Ecol Evol **32**, 948-963 (2017).*

18. *Jost L. GST and its relatives do not measure differentiation. Mol Ecol **17**, 4015-4026 (2008).*
