# Supplementary material for: Fragmentation by major dams and implications for the future viability of platypus populations
Source: Commun Biol. 2022 Nov 3;5:1127. doi: 10.1038/s42003-022-04038-9 (PMC9633709; doi:10.1038/s42003-022-04038-9)
Supplement: Supplementary file 3 — Description of Additional Supplementary Files [file 42003_2022_4038_MOESM3_ESM.pdf]

Description of Additional Supplementary Files

File name: Supplementary Data 1

Description: 3D PCA of Central New South Wales Rivers

File name: Supplementary Data 2

Description: 3D PCA of Border Rivers

File name: Supplementary Data 3

Description: 3D PCA of Upper Murray Rivers

File name: Supplementary Data 4

Description: 3D PCA of Snowy Rivers
