# Supplementary material for: Fragmentation by major dams and implications for the future viability of platypus populations
Source: Commun Biol. 2022 Nov 3;5:1127. doi: 10.1038/s42003-022-04038-9 (PMC9633709; doi:10.1038/s42003-022-04038-9)
Supplement: Supplementary file 8 — Reporting Summary [file 42003_2022_4038_MOESM8_ESM.pdf]

Corresponding author(s): Luis Mijangos

Last updated by author(s): Aug 30, 2022

Reporting Summary

Nature Research wishes to improve the reproducibility of the work that we publish. This form provides structure for consistency and transparency in reporting. For further information on Nature Research policies, see our Editorial Policies and the Editorial Policy Checklist.

Statistics

For all statistical analyses, confirm that the following items are present in the figure legend, table legend, main text, or Methods section.

n/a	Confirmed	
☐	☒	The exact sample size (*n*) for each experimental group/condition, given as a discrete number and unit of measurement
☐	☒	A statement on whether measurements were taken from distinct samples or whether the same sample was measured repeatedly
☐	☒	The statistical test(s) used AND whether they are one- or two-sided<br>*Only common tests should be described solely by name; describe more complex techniques in the Methods section.*
☒	☐	A description of all covariates tested
☐	☒	A description of any assumptions or corrections, such as tests of normality and adjustment for multiple comparisons
☐	☒	A full description of the statistical parameters including central tendency (e.g. means) or other basic estimates (e.g. regression coefficient) AND variation (e.g. standard deviation) or associated estimates of uncertainty (e.g. confidence intervals)
☒	☐	For null hypothesis testing, the test statistic (e.g. *F*, *t*, *r*) with confidence intervals, effect sizes, degrees of freedom and *P* value noted<br>*Give P values as exact values whenever suitable.*
☒	☐	For Bayesian analysis, information on the choice of priors and Markov chain Monte Carlo settings
☒	☐	For hierarchical and complex designs, identification of the appropriate level for tests and full reporting of outcomes
☒	☐	Estimates of effect sizes (e.g. Cohen's *d*, Pearson's *r*), indicating how they were calculated

Our web collection on statistics for biologists contains articles on many of the points above.

Software and code

Policy information about availability of computer code

Data collection	Samples were genotyped using DArTseqTM (DArT Pty Ltd, Canberra, ACT, Australia). DArT's procedure uses a combination of genome complexity reduction methods using restriction enzymes, implicit fragment size selection and next-generation sequencing to produce thousands of SNPs randomly distributed throughout the genome (Kilian et al., 2012). Read sequences were processed using proprietary DArT analytical pipelines (Kilian et al., 2012) and mapped to the representative platypus genome (mOrnAna1.p.v1, GenBank assembly accession: GCA_004115215.2; total sequence length of 1.8 Gbps, 305 scaffolds with an N50 of 83 Mbp). Refer to Georges et al. (2018) for details of DArT sequencing, genotyping and filtering processes.
Data analysis	The programs used to analyse the data were: R package Hierfstat 0.5-11, R package dartR 2.0.4 and R version 4.2.1.<br>The R scripts used for this research work are stored in GitHub: https://github.com/mijangos81/Platypus and have been archived within the Zenodo repository: https://doi.org/10.5281/zenodo.7039778.

For manuscripts utilizing custom algorithms or software that are central to the research but not yet described in published literature, software must be made available to editors and reviewers. We strongly encourage code deposition in a community repository (e.g. GitHub). See the Nature Research guidelines for submitting code & software for further information.

Data

Policy information about availability of data

All manuscripts must include a data availability statement. This statement should provide the following information, where applicable:

- Accession codes, unique identifiers, or web links for publicly available datasets
- A list of figures that have associated raw data
- A description of any restrictions on data availability

The datasets used for this research work are stored in GitHub: https://github.com/mijangos81/Platypus and have been archived within the Zenodo repository: https://doi.org/10.5281/zenodo.7039778.

Field-specific reporting

Please select the one below that is the best fit for your research. If you are not sure, read the appropriate sections before making your selection.

☐ Life sciences ☐ Behavioural & social sciences ☒ Ecological, evolutionary & environmental sciences

For a reference copy of the document with all sections, see nature.com/documents/nr-reporting-summary-flat.pdf

Ecological, evolutionary & environmental sciences study design

All studies must disclose on these points even when the disclosure is negative.

Study description	To determine whether major dams have reduced dispersal and gene flow between platypus groups, we analysed genetic data from platypuses sampled in nine rivers; five rivers were regulated by major dams, and four were unregulated (Fig. 1). If major dams adversely affected gene flow between platypus groups, we predicted the following: a) individuals and groups separated by a major dam in a river should be more differentiated than in an unregulated river, and; b) genetic differentiation across major dams should correlate with the time since the dam was built.
Research sample	A total of 295 platypuses were captured and blood sampled across four river regions in southeast Australia. Details of the platypuses that were sampled such as sex, age class, weight and location (longitude and latitude) can be found in Supplementary Table 2.
Sampling strategy	We aimed to cover a minimum of 40 km of each unregulated river and 20 km of river above and below major dams on regulated rivers.
Data collection	Sampling for microsatellites in Central NSW is described in Kolomyjec et al. (2008, 2009). For SNPs at all other sites, we aimed to cover a minimum of 40 km of each unregulated river and 20 km of river above and below major dams on regulated rivers. The procedure of trapping and sampling platypuses, including details of anaesthesia, used in this study have been described elsewhere (Bino et al., 2018; Hawke, Bino, & Kingsford, 2021b). Briefly, platypuses were captured using fyke nets or unweighted mesh (gill) nets and implanted with a Passive Integrated Transponder (PIT) tag (Trovan) to identify recaptured individuals. Platypuses were then weighed, measured, sexed, aged, and blood collected (~2 ml) and stored in Qiagen RNAprotect® animal blood tubes (Qiagen, Hilden, Germany). Gilad Bino, Tahneal Hawke, and Tom Grant carried out fieldwork and collected samples.
Timing and spatial scale	Platypuses were surveyed for 172 nights (1,550 net hours) across 108 sites (Jan 2016- May 2018, Fig. 1), aiming to cover a minimum of 40 km of each unregulated river and 20 km of river above and below dams on regulated rivers.
Data exclusions	Two samples, each collected in a different river (V30 in Ovens and V32 in Mitta Mitta), showed contrasting genetic patterns relative to samples collected in the same river (Supplementary Figure 1). Relatedness analyses performed in the R package related (Pew et al., 2015) revealed these two samples had closer relatives in the opposite river (Supplementary Table 1). Additionally, the locations of these two samples were separated by 46 Km, steep mountainous terrain, and a river system. Under these conditions, we considered that dispersal events were unlikely and concluded that samples were mislabelled and therefore assigned them to the presumed correct river and site. Relatedness analyses also identified two pairs of samples in which each pair was collected from the same individual (i.e., recaptures; samples T3-T5 and T28-T42; Supplementary Table 1). Consequently, we removed one sample from each pair. In the unlikely event that these were pairs of identical twins, it would still be appropriate to remove one of each pair.
Reproducibility	Platypuses were collected during the field study over two years, and were not repeated as in experiments. We described the methods of data collection and analyses in detail, allowing the work to be repeated.
Randomization	We assigned individuals a priori into groups, based on the river that individuals were sampled and whether they were sampled below or above major dams.
Blinding	Blinding was not applicable to this study since the geographic and temporal metadata associated with each sample was key to carry research.

Did the study involve field work? ☒ Yes ☐ No

Field work, collection and transport

Field conditions	Platypus were captured in small, shallow streams (<1m). Nets were set in the late afternoon and checked every 3 hours until shortly

Field conditions	after sunrise. Mesh nets were used in large (>50m) and deep (>1-2m) pools, with the net parallel to the riverbank. Nets were set from dusk until 01.00 AM, checking them every 2-3 minutes with a spotlight and removing platypuses and non-target species immediately.
Location	The location of each sampled platypus is reported in Supplementary Table 2.
Access & import/export	Trapping and handling of platypuses for the SNP samples were carried out in accordance with guidelines and approved by the NSW Office of Environment and Heritage (SL101655), NSW Department of Primary Industries (P15/0096-1.0 & OUT15/26392), and UNSW's Animal Care and Ethics Committee (16/14A). Trapping and handling of platypuses for the microsatellite samples from the Nepean and Wingecarribee Rivers was approved under the [then] NSW Department of Environment and Climate Change Scientific Research License # S10478, NSW Department of Primary Industries (DPI) Scientific Research Permit #F84.1245 and NSW DPI Animal Research Authority - Trim File No. 01/1091. Consent to enter Special Metropolitan [catchment] Areas was provided by the [then] Sydney Catchment Authority (Reference No. 3008/07934; 2009).
Disturbance	Disturbance to the sampled platypuses was minimized by checking Fyke nets every three hours and mesh nets every three minutes with a spotlight and removing platypuses and non-target species immediately. Captured platypuses were transferred from nets to pillow cases where they were kept until processed, following established protocols (Bino, Kingsford, Grant, Taylor, & Vogelnest, 2018). Individuals were placed in an induction chamber and anaesthetized over 5-7 minutes, using isoflurane (Pharmachem, 5%) in oxygen (3 L/min; Chinnadurai, Strahl-Heldreth, Fiorello, & Harms, 2016; Fiorello, Harms, Chinnadurai, & Strahl-Heldreth, 2016). Anaesthesia was maintained using a T-piece facemask, with isoflurane (1.5%) in oxygen (1.0 L/min; Vogelnest & Woods, 2008).

Reporting for specific materials, systems and methods

We require information from authors about some types of materials, experimental systems and methods used in many studies. Here, indicate whether each material, system or method listed is relevant to your study. If you are not sure if a list item applies to your research, read the appropriate section before selecting a response.

Materials & experimental systems

n/a	Involved in the study
☒	☐ Antibodies
☒	☐ Eukaryotic cell lines
☒	☐ Palaeontology and archaeology
☐	☒ Animals and other organisms
☒	☐ Human research participants
☒	☐ Clinical data
☒	☐ Dual use research of concern

Methods

n/a	Involved in the study
☒	☐ ChIP-seq
☒	☐ Flow cytometry
☒	☐ MRI-based neuroimaging

Animals and other organisms

Policy information about studies involving animals; ARRIVE guidelines recommended for reporting animal research

Laboratory animals	The study did not involve laboratory animals.
Wild animals	A total of 295 platypuses were captured and blood sampled across four river regions in southeast Australia. Platypuses were captured using fyke nets or unweighted mesh (gill) nets and implanted with a Passive Integrated Transponder (PIT) tag (Trovan) to identify recaptured individuals. Platypuses were then weighed, measured, sexed, aged, and blood collected (~2 ml). Details of the platypuses that were sampled such as sex, age class, weight and location (longitude and latitude) can be found in Supplementary Table 2.
Field-collected samples	The study did not involve samples collected from the field.
Ethics oversight	Trapping and handling of platypuses for the SNP samples were carried out in accordance with guidelines and approved by the NSW Office of Environment and Heritage (SL101655), NSW Department of Primary Industries (P15/0096-1.0 & OUT15/26392), and UNSW's Animal Care and Ethics Committee (16/14A). Trapping and handling of platypuses for the microsatellite samples from the Nepean and Wingecarribee Rivers was approved under the [then] NSW Department of Environment and Climate Change Scientific Research License # S10478, NSW Department of Primary Industries (DPI) Scientific Research Permit #F84.1245 and NSW DPI Animal Research Authority - Trim File No. 01/1091. Consent to enter Special Metropolitan [catchment] Areas was provided by the [then] Sydney Catchment Authority (Reference No. 3008/07934; 2009).

Note that full information on the approval of the study protocol must also be provided in the manuscript.
